# Overlap of Granulomatosis With Polyangiitis and IgA Nephropathy

**DOI:** 10.7759/cureus.18672

**Published:** 2021-10-11

**Authors:** Bassem S Zeidan, Andrea Hernandez, Parth Desai

**Affiliations:** 1 Internal Medicine, HCA Medical Center of Trinity, Trinity, USA; 2 Internal Medicine, Medical Center of Trinity, Trinity, USA; 3 Critical Care Medicine, Medical Center of Trinity, Trinity, USA

**Keywords:** necrotizing and crescentic glomerulonephritis, wegners granulamatosis, therapeutic plasmapheresis, iga glomerulonephritis, c anca, iga nephropathy, granulomatosis with polyangiitis, acute glomerulonephritis

## Abstract

Acute glomerulonephritis is a constellation of renal disorders which are precipitated by an immunologic mechanism triggering inflammation and proliferation of glomerular tissue resulting in damage to the basement membrane, mesangium, or capillary endothelium. Two of the most well-known manifestations of glomerulonephritis are granulomatosis with polyangiitis (GPA) and IgA nephropathy (IgAN). To our knowledge, these diseases are often found separately and are rarely diagnosed in the same patient. Here, we discuss a case of a 35-year-old male who presented and was diagnosed with simultaneous GPA and IgAN. His renal biopsy was significant for extensive, crescentic, and necrotizing glomerulonephritis and IgA staining on immunofluorescence indicating severe renal damage. Despite full immunosuppressive therapy, our patient failed to recover his renal function. In our case, we hope to raise awareness of these disorders and early recognition of the clinical features. We believe that this case would prompt providers to pursue diagnostic workups to detect these diseases early on, especially when symptoms progressively worsen and become systemic. As demonstrated in our patient, delay in diagnosis can lead to irreversible damage to a patient’s renal function, especially when two types of glomerulonephritis are present.

## Introduction

Acute glomerulonephritis is characterized by the abrupt onset of hematuria often accompanied by proteinuria, hypertension, edema, and renal dysfunction [1]. There are many causes of acute glomerulonephritis including post-infectious, autoimmune, and hereditary etiologies. The most common glomerulonephritis globally is IgA nephropathy (IgAN), which is usually preceded by respiratory and gastroenteritis infections. It is characterized as IgA immune-complex deposition in glomerular mesangial cells results in its proliferation [2]. Another form of acute glomerulonephritis well known but rare is granulomatosis with polyangiitis (GPA), formally known as Wegener's granulomatosis. GPA is characterized as a systemic pauci-autoimmune vasculitis that typically involves the upper respiratory tract, lower respiratory tract (bronchi and lung), and kidney, with varying degrees of disseminated vasculitis [3]. We would like to discuss a rare case of GPA and IgAN that were concurrently diagnosed at the time of presentation.

## Case presentation

We present a case of a 35-year-old Caucasian male who was admitted to the hospital for complaints of lower extremity swelling over one week. The patient also noticed that his urine had become foamy, tea-colored and urine output had decreased despite hydrating adequately. Five months prior to presentation, the patient began experiencing sinusitis-like symptoms described as sinus congestion, postnasal drip, facial swelling, and frontal head pressure. This was associated with progressive weakness, easy fatigability, lethargy, and migratory joint pain predominantly involving the left shoulder and hips. He reported taking two to three tablets of over-the-counter ibuprofen at least every other day for the past three to four weeks to relieve the sinus pain. In addition, two months prior to presentation, he was treated with a 10-day course of amoxicillin/clavulanic acid, levofloxacin, and a five-day regimen of prednisone without any relief. As symptoms progressed, he underwent computed tomography (CT) scan of the sinuses, revealing nasal polyps. He subsequently followed up with an ear nose throat (ENT) doctor and then underwent an in-office cauterization to remove the nasal polyps which he reported did mildly improve his sinusitis-like symptoms. He was given a prescription for a 10-day regimen of clindamycin and another regimen of prednisone, both of which he completed approximately 10 days prior to being seen at our facility.

Due to minimal improvement, he underwent outpatient laboratory tests one week before arrival, which was notable for acute kidney injury with blood urea nitrogen (BUN) elevated at 37 mg/dL and creatinine of 3.6.mg/dL. His albumin was minimally low at 3.1 g/dL with a normal globulin of 3 g/dL. Aspergillus antibodies were done and found to be negative. An antistreptolysin was also negative as he reported streptococcal exposure from his son at the time. His lipase was negative. His white blood cell count (WBC) was also within the normal range. He was noted to be anemic with hemoglobin of 10.6 g/dL and hematocrit of 32% with normal limits. Platelets were normal at 234,000 per mcL. Urinalysis revealed cloudy, brown-red-colored urine that was negative for ketones. Specific gravity was 1.015, pH was 5.0 and protein was greater than 300 mg/dL. Over the next couple of days, the patient had increased pain especially in his left shoulder and both hips but the pain in his left shoulder was so severe that he was barely able to move it. For this reason, he went back to his primary care provider the day prior to hospitalization, and more blood work was ordered which was notable for rapidly worsening renal function. Repeat labs showed a BUN 76 g/dL and creatinine of 12 g/dL. His sodium had dropped to 119 mmol/L and chloride was 88 mmol/L with a carbon dioxide (CO_2_) of 15 mmol/L. Phosphorus level was elevated to 7.8 mmol/L, albumin had dropped even further to 2.5 g/dL. His white count was elevated to 15,000 x 10^9^/L and he remained anemic. This prompted instruction to come to the hospital for further management.

A day later, he was hemodynamically stable on arrival to the hospital, but metabolic panels were significant for multiple disturbances (see Table 1). It showed a BUN 97 mg/dL, and creatinine of 14.1 mg/dL with GFR less than 15 mL/min. Additionally, he had a bicarbonate level of 15 mmol and uric acid of 11.4 mg/dL with an anion gap of 18 mmol/L, indicating elevated anion gap metabolic acidosis in addition to worsening sodium levels of 117 mmol/L and myoglobin level 274 mg/mL. He was subsequently admitted to the intensive care unit (ICU) in order to undergo emergent hemodialysis. Further workup was significant for a C-reactive protein level of 241 mg/L, erythrocyte sedimentation rate (ESR) of 81 mm/hr. HIV and hepatitis testing were negative. Serum C-antinuclear cytoplasmic autoantibodies (C-ANCA) level was >8.0 AI. C-ANCA antibodies are highly sensitive and specific to GPA. As GPA was suspected, high dose intravenous (IV) methylprednisolone, and plasmapheresis plus hemodialysis and renal biopsy were promptly performed. Renal biopsy was significant for extensive, crescentic, and necrotizing glomerulonephritis in 27/29 Glomeruli consistent with ANCA-mediated glomerulonephritis on light microscopy. On immunofluorescence, there was mesangial staining for IgA, which met the criteria IgAN. Once the biopsy was confirmed, he was initiated on IV rituximab therapy to treat GPA and was transitioned to high dose oral prednisone which was eventually tapered down. His urine output mildly improved but there was minimal improvement in renal function. On one month follow-up, his renal function failed to improve and his C-ANCA titers remained elevated at 6.9. He was tried on cyclophosphamide which also failed. Unfortunately, the patient remained on scheduled hemodialysis despite these immunosuppressive therapies and was referred for renal transplant four months later.

**Table 1:** Timeline of metabolic disturbances

Metabolic Panel	Glomerular Filtration Rate mL/min	Blood Urea Nitrogen mL/dL	Creatinine mg/dL	Sodium mmol/L	Bicarbonate mmol/L	Phosphorus m mol/L	Uric Acid mg/dL	Albumin g/dL
One week prior to admission	<15	37	3.6	137	24	Not available	Not available	3.1
One day prior to admission	<15	76	12.0	119	15	7.8	Not available	2.5
Day of admission	<15	97	14.1	117	11	10.2	11.4	2.0

## Discussion

GPA is a devastating systemic autoimmune disorder characterized as a pauci-immune vasculitis involving the upper and lower respiratory tract, and kidneys, with varying degrees of disseminated vasculitis. Patients with GPA usually present with nonspecific symptoms of generalized systemic illness including fever, malaise, weight loss, arthralgia, and myalgia [4]. Similar to our patient, the earliest complaint is often sinusitis-like symptoms. These earliest complaints, which are also the most common reasons for seeking medical attention, are usually related to upper respiratory tract problems including sinus pain, purulent nasal discharge, epistaxis, nasal ulceration, and serous otitis media [5]. IgAN, characterized by mesangial IgA deposition, is the most common type of glomerulonephritis worldwide. It is preceded by respiratory and gastrointestinal infections and presents with macroscopic hematuria in about 40% to 45% of patients, with microscopic hematuria and proteinuria in about 35% to 40%, and with nephrotic syndrome or acute renal failure in the remainder [6].

The relationship between GPA and IgAN is unclear and underreported. No more than 30 cases with IgAN and coexisting IgG-ANCA positivity have been reported to date, mainly case reports and two case series [7]. C-ANCA is frequently observed in patients with GPA which primarily target proteinase 3 (PR3), a serine protease expressed in azurophilic granules of neutrophils. Serum anti-PR3 antibodies produce a cytoplasmic staining pattern on immunofluorescence assays, explaining the designation C-ANCA [8]. When activated, neutrophils display the majority of granular PR3 on their cell-surface membranes which then bind to the surface of primed neutrophils and activate various functions such as the oxidative burst [9]. This C-ANCA mediated effect also causes activation of IgG Fc receptors (FcRs) namely FCGR2A (CD32A) and FCGR3B (CD16B) by anti-PR3 antibodies [10]. Activation of these receptors often leads to stronger phagocytosis, respiratory burst, and neutrophil degranulation. Similarly, IgA's pro-inflammatory and anti-inflammatory effects are determined through engagement with the FcR, which may lead to an IgA-ANCA synergistic effect of disease activity and severity in GPA [8]. Limited but available evidence suggests that ANCA-positive patients with IgAN frequently present with rapidly decreasing kidney function and extrarenal manifestations have more serious histological lesions, with a higher percentage of crescentic glomeruli and a higher degree of tubular atrophy, compared with ANCA-negative patients [11]. This is demonstrated in our patient given the involvement of 27/29 glomeruli and subsequent irreversible damage to his kidneys despite adequate immunosuppressive therapy.

## Conclusions

Two of the most well-known manifestations of glomerulonephritis are GPA and IgAN. The relationship between GPA and IgAN is very rare and both conditions can often be overlooked. Limited but available evidence suggests that ANCA-positive patients with IgAN frequently present with rapidly decreasing kidney function and extrarenal manifestations resulting in a devastating impact on the patient. Delay in diagnosis can lead to irreversible damage to a patient’s renal function, especially when two types of glomerulonephritis are present. Our case highlights the importance of recognizing these clinical manifestations and prompt investigation early in the disease course.
